# Supplementary material for: Towards personalized care: Factors associated with the quality of life of residents with dementia in Australian rural aged care homes
Source: PLoS One. 2020 May 21;15(5):e0233450. doi: 10.1371/journal.pone.0233450 (PMC7241691; doi:10.1371/journal.pone.0233450)
Supplement: S1 File — Interview questions for aged care manager/staff. (DOCX) [file pone.0233450.s001.docx]

**S1 Interview guide**

**Interview questions for aged care manager/staff**

These questions are provided as a guide for the Interviews. The discussion will not be limited to these questions. The interview will be audiotaped with participant consent and transcribed for analysis and reporting purposes

Welcome – Introduction and consent

1. Think back over all the two months you have participated in the program and tell us what was most useful -
2. to the resident and their family members;
3. to you; and
4. to the residential home.
5. How would you like to define person-centred dementia care?
6. Give me a picture of how the current care assessment and plan are designed? Explain the influence of current care plans on the elderly persons overall health. Please provide examples relating to physical and mental healthcare and well-being.
7. What are the changes you expect in care-plans for people with dementia? How Harmony in the Bush program contributed in developing a person-centred care plan and improving health and well-being of the residents?
8. Please tell me about your communications with residents with dementia and other staff in support a resident? How would you describe your role in care-related decision making?
9. Please tell me about your participation in education and training programs on dementia care. Do you get enough support from your supervisor and other staff on the floor?
10. Please tell me about your work-related stress, satisfaction and health & well-being? Have you seen any changes after harmony in the Bush program?
11. What aspects of organisational culture restrict you in providing cate to a resident with dementia? Please tell me the contribution of Harmony in the Bush program in building relationships among and between staff and residents.
12. How would you define the organisational capacity in implementing personalised dementia care? What changes/additions would make the organisation better? any other comments?

I think we’ve come to the end of our questions. Let me say thank you for your honest opinions. We really appreciate your help.
