# Supplementary material for: Towards personalized care: Factors associated with the quality of life of residents with dementia in Australian rural aged care homes
Source: PLoS One. 2020 May 21;15(5):e0233450. doi: 10.1371/journal.pone.0233450 (PMC7241691; doi:10.1371/journal.pone.0233450)
Supplement: S2 File — Questions and discussion points. (DOCX) [file pone.0233450.s002.docx]

**S2 Focus group schedule**

**Questions and discussion points**

These questions and discussion points are provided as a guide for the FGD. The discussion will not be limited to these questions. The focus groups will be audiotaped with participant consent and transcribed for analysis and reporting purposes.

Moderator: Hello and Welcome to this group discussion. My name is [insert name of moderation] and I am here working as the facilitator/moderator. I am working on behalf of researchers [or I am working on a research study] at Flinders University. My role is to help get a conversation going and to make sure we cover a number of important topics that we would like your input on.

Introductions

Purpose: First of all, I would like to thank you all for taking time out of your day to come here and discuss your ideas. The overall goal is to hear your thoughts about Harmony in the Bush Program which you have all participated in over the last two months. In particular, we are interested in your views about the usefulness of the program to you and your organisation.

Explaining the purpose for setting up the focus group meeting:

o You are the experts and we are here to learn from you

o This is strictly voluntary

Housekeeping:

The total length of time of the focus group meeting is expected to be about [insert expected duration].

As far as the focus groups are concerned, there are a few “ground rules”

• I might move you along in conversation. Since we have limited time, I’ll ask that questions or comments off the topic be answered after the focus group session

• I’d like to hear everyone speak so I might ask people who have not spoken up to comment

• Please respect each other’s opinions. There’s no right or wrong opinion or answer. We want to hear what each of you think and it’s okay to have different opinions.

• We’d like to stress that we want to keep the sessions confidential, so we ask that you not use names or anything directly identifying when you talk about your experiences. We also ask that you not discuss other participants’ responses outside of the discussion. However, because this is in a group setting, the other individuals participating will know your responses to the questions and we cannot guarantee that they will not discuss your responses outside of the focus group.

**Do you have any questions so far?**

Again, your participation here today is totally voluntary. So, if you are okay with moving forward, we would like to get your consent.

1. Think back over all the two months you have participated in the program and tell us what was most useful -
2. to the resident and their family members;
3. to you; and
4. to the residential home.
5. How would you like to define person-centred dementia care?
6. Give me a picture of how the current care assessment and plan are designed? Explain the influence of current care plans on the elderly persons overall health. Please provide examples relating to physical and mental healthcare and well-being.
7. What are the changes you expect in care-plans for people with dementia? How Harmony in the Bush program contributed in developing a person-centred care plan and improving health and well-being of the residents?
8. Please tell me about your communications with residents with dementia and other staff in support a resident? How would you describe your role in care-related decision making?
9. Please tell me about your participation in education and training programs on dementia care. Do you get enough support from your supervisor and other staff on the floor?
10. Please tell me about your work-related stress, satisfaction and health & well-being? Have you seen any changes after harmony in the Bush program?
11. What aspects of organisational culture restrict you in providing cate to a resident with dementia? Please tell me the contribution of Harmony in the Bush program in building relationships among and between staff and residents.
12. How would you define the organisational capacity in implementing personalised dementia care? What changes/additions would make the organisation better? any other comments?

I think we’ve come to the end of our questions. Let me be the first to say thank you for your honest opinions – you were tremendously helpful at this very early, but very important stage. Again, thank you very much for your participation today. We really appreciate your help.
