# Supplementary material for: Towards personalized care: Factors associated with the quality of life of residents with dementia in Australian rural aged care homes
Source: PLoS One. 2020 May 21;15(5):e0233450. doi: 10.1371/journal.pone.0233450 (PMC7241691; doi:10.1371/journal.pone.0233450)
Supplement: Attachment — Submitted filename: Response to Reviewers_PONE-D-20-03933.docx [file pone.0233450.s003.docx]

To

Tim Luckett, Academic Editor

PLOS ONE

RE: Revised manuscript “Towards personalized care: Factors associated with the quality of life of residents with dementia in Australian rural aged care homes” [PONE-D-20-03933]

Dear Dr Lackett,

Thank you for the opportunity to strengthen our manuscript submission. We found the reviewer’s comments helpful and have addressed them in the point-by-point table below.

Please do not hesitate to contact me should you have any queries to the revision.

We thank you for your time and look forward to hearing from PLOS ONE in regard to our manuscript.Top of Form

A point-by-point response letter has been accompanied with our revised manuscript with track changes and final manuscript. This letter provides a detailed response to each reviewer/editorial point raised, describing exactly what amendments have been made to the manuscript text and where these can be viewed. All changes to the manuscript are indicated in the text by highlighting or using track changes.

Kind Regards

Dr Mohammad Hamiduzzaman

Research Fellow

Flinders University Rural Health SA

College of Medicine & Public Health

Flinders University

| **Manuscript revision** | **Changes made** | **Track-changes location in manuscript** |
| --- | --- | --- |
| **Journal requirements** |  |  |
| 1. Please ensure that your manuscript meets PLOS ONE's style requirements, including those for file naming. | Title page and manuscript body have been formatted according to the journal guidelines | Throughout the manuscript where needed. |
| 2. Please ensure you have thoroughly discussed any potential limitations of this study within the Discussion section.  In addition, please include additional information regarding the interview guide used in the study and ensure that you have provided sufficient details that others could replicate the analyses. For instance, if you developed a guide as part of this study and it is not under a copyright more restrictive than CC-BY, please include a copy, in both the original language and English, as Supporting Information. | The non-participation of family members of RwD in interviews and focus groups is a limitation, as is the qualitative study, perspectives of one participant group does not allow the findings to be comprehensive. However, the richness in the findings originates from the insight of a good number of staff who have been working in rural aged care homes for a long time, especially in diverse cultural and organizational contexts. The willingness of the staff in sharing their stories with RwD and the examples they provide contribute to understanding the dimensions of personalized dementia care. Another potential limitation could be social desirability bias. The participants may have answered questions in a manner that will be viewed positively by researchers. The participants were given adequate information on consent and confidentiality of the data and the opportunity to withdraw from the study at any time point. However, it may be possible the staff who had negative or alternate views did not volunteer to participate in the interview and focus group. Another limitation is that we do not explore the factors in providing personalized care in terms of the centers’ size, number of residents and classification of rural locations that may have potential impact on transferability of findings. Finally, the study did not include participant observation and that the care dimensions were derived only from interviews with the participants. These are the areas that require attention in further investigation to understand the dimensions and nature of factors involved in personalized dementia care implementation, particularly in rural facilities.  Interview guide and focus groups schedule are attached as supporting information | Discussion ˃  Limitation:  Page no. 25 (Line No. 1-16)  Page 29 - 32 |
| **Reviewers’ comments** |  |  |
| **Reviewer 1** |  |  |
| 1. Is the manuscript technically sound, and do the data support the conclusions?  The manuscript must describe a technically sound piece of scientific research with data that supports the conclusions. Experiments must have been conducted rigorously, with appropriate controls, replication, and sample sizes. The conclusions must be drawn appropriately based on the data presented.  Reviewer #1: Partly | **Conclusion**  The dimensions associated with the quality of personalized dementia care in rural aged care homes emerged from the data provided by care staff in five rural aged care homes in Queensland and South Australia, Australia. Personalized dementia care is multidimensional and can be described using a conceptual framework that incorporates dialectical relationships and events occur in aged care homes. To achieve quality care, the dimensions that are of importance for the aged care homes include resident and family centeredness, assessment and care planning, staff education and training, staff-resident interaction, work-life balance, leadership and organizational culture and physical environment and safety.  The current service delivery focusses too much on perceived generalized clinical care needs rather than specific person-centered care. The inclusion of the voice of residents and their family members in designing care plan is in need to establish personalized care practice for RwD to give them opportunity to live with a control over care service. Emphasis on the staff retention and recruitment and resource adequacy is important to improve quality of personalized dementia care in rural aged care homes. The aged care policy makers and organization leaders should consider work-life balance of staff as a key issue and respond with mentorship and formal support structures.  The study’s findings will contribute to the knowledge of dimensions of personalized dementia care. It is evident that rural aged care homes require additional commitment from the policy makers relating to resource stabilization and improvement. This study confirms the need for a holistic approach to respond to the barriers and integrate personalized care to improve the quality of life of RwD in rural aged care homes. | Conclusion  Page No. 25 (Line No – 18-35); 26 (Line No. 1-2) |
| 2. Has the statistical analysis been performed appropriately and rigorously?  Reviewer #1: N/A | Not applicable | Not applicable |
| 3. Have the authors made all data underlying the findings in their manuscript fully available?  The [PLOS Data policy](http://www.plosone.org/static/policies.action#sharing) requires authors to make all data underlying the findings described in their manuscript fully available without restriction, with rare exception (please refer to the Data Availability Statement in the manuscript PDF file). The data should be provided as part of the manuscript or its supporting information, or deposited to a public repository. For example, in addition to summary statistics, the data points behind means, medians and variance measures should be available. If there are restrictions on publicly sharing data—e.g. participant privacy or use of data from a third party—those must be specified.  Reviewer #1: Yes | Not applicable | Not applicable |
| 4. Is the manuscript presented in an intelligible fashion and written in standard English?  PLOS ONE does not copyedit accepted manuscripts, so the language in submitted articles must be clear, correct, and unambiguous. Any typographical or grammatical errors should be corrected at revision, so please note any specific errors here.  Reviewer #1: Yes | Not applicable | Not applicable |
| 5A. Reviewer #1: Thank you for the opportunity to review this manuscript exploring factors associated with the quality of life of residents with dementia in Australian rural aged care homes. This is a valuable topic and the need for research in this area and aims of this study are well-defined. I do have some concerns about the presentation of results, which I feel could be more clearly articulated and linked back to the theoretical framework noted in the methods section. I have listed more specific points below for your consideration that I hope will be helpful.  • Aims presented clearly in the abstract and it is evident to readers that this paper reports qual findings of the larger mixed-methods study. | Not applicable | Not applicable |
| 5B. Further information about recruitment is needed – it is noted that managers of each site were contacted to recruit the aged care workers, but what measures were taken to avoid potential feelings of coercion? How were the workers invited to participate in the study and how was the information sheet and consent form provided to potential participants? Was this in person? Email? And by whom? More detail needed. | An Information sheet and consent form were emailed to the facility managers for distribution among staff. Participants were free to contact the investigators if they wished to be interviewed and or to participate in focus group. They were also informed that they could choose to withdraw at any time of the project without adverse effects on their immediate or future care provision. | Materials and methods ˃ Data collection; Page No. 7 (line No. 7-11) |
| 5C. Please check the English and sentence structure throughout the manuscript. e.g. ‘Some family members were labelled…’  or ‘… intervenes a non-pharmacological personalised dementia care model...’ - what does this mean? | Some family members were blamed if they made a complaint or were too demanding whilst others were afforded respect and care.  … trials a personalized dementia care program.  Checked English and sentence structure throughout the manuscript and revised where necessary. | Findings ˃Dimensions of personalized care ˃ Residents and family centredness; Page No. 10 (Line No. 42-43)  Methods and materials  Page No. 6 (Line No. 9-10) |
| 5D. It is very unclear how many participants were involved in this qualitative portion of the study. Participant characteristics were presented for the entire cohort which may or may not reflect the characteristics of the qualitative sub-sample. It would be good to present demographic information about the interviewees/focus group participants only so we know whose perspectives are being represented in this paper. | One hundred and four care staff participated in interviews and or focus groups at three phases: post-intervention [interview: 38; focus group: 13], one-month follow-up [interview: 11; focus group: 2] and three-months follow-up [interview: 16; focus group: 5].  A total number of 104 staff participated, and of them 84% were female. | Methods and materials ˃ Data collection  Page No. 7 (Line No. 18-21)  Findings ˃ Baseline demographics  Page No. 8 (Line No. 14-15) |
| 5E. A major concern is that quotes don’t seem to support what is being said in the text in every instance. E.g. do the quotes on pg. 7 really illustrate ‘excluding’ family member’s perspectives? Please ensure quotes are supporting of themes or that further explanation in-text is included to provide clarity about how quotes relates to the theme. | *… we see their* [residents] *family members visit here and meet their parents on different occasions such as celebrating birthdays. Some families come in once or twice every month, some do not visit very often. We discuss with family members [legal guardian] for each resident’s medical history at admission time, but I do not see continuous engagement of staff with residents’ family members* [in providing care]*.* (Staff, ACH 4 – One-month follow-up interview) | Findings ˃ Dimensions of personalized dementia care  Page No. 10 (Line No. 11-15) |
| 5F. All quotes can be condensed to enhance readability. | All the quotes are checked. Quotes are either elaborated or condensed throughout the manuscript where possible to enhance readability. | Findings ˃ Dimensions of personalized dementia care  Page No. 10, 11, 16, 17 |
| 5G. Interviews and focus groups were conducted at different time points…does this add to these findings in any way? | One hundred and four care staff participated in interviews and or focus groups at three phases: post-intervention [interview: 38; focus group: 13], one-month follow-up [interview: 11; focus group: 2] and three-months follow-up [interview: 16; focus group: 5].  Time points of interviews and focus groups are added in all quotes throughout the findings section, such as,  *Yeah, because if you don’t, you know, you need that communication all the time, because we’re here for the residents, not ourselves, so, you know …* (Staff, ACH 5 – One-month follow-up focus group ) | Methods and materials ˃ Data collection  Page No. 7 (Line No. 18-21)  Findings ˃ Dimensions of personalized dementia care  Page No. 9-20 |
| 5H. Please write out acronyms used in the first instance in full (quote on pg. 11) | When we have a new staff member come, who apparently is quite well educated on the whole progressively lowered stress threshold, so you take their ideas on board and then just making sure that everyone who is here, remains on board with it. (Staff, ACH 3) | Findings ˃ Dimensions of personalized dementia care  Page No. 17 (Line No. 9-10) |
| 5I. Please ensure font size for all text in the figures is consistent – ‘Personalised care plan in place’ | Reviewed and make the font size consistent  Change in figure: Personalized dementia care plan in place | Discussion  Page No. 22  Discussion Page No. 22 |
| 5J. Given the theoretical background presented in the methods section, I expected to see further discussion around how the results fit/did not fit with the theoretical framework. | The study’s findings indicate the personalized dementia care needs more attention in rural aged care homes in Australia, and provide insight into resident and family, staff and organization spheres that are of importance in planning and practicing person-centered care for the RwD (Fig. 1). The exploration of factors under these spheres is influenced by the multidimensional nursing home care model developed by Rantz et al. (1998), who emphasize on operational resources and components, such as central focus, interactions, milieu, environment, care, staff and safety. The factors and associated realities emerged in this study contribute to the contextualization of the dimensions based on dynamic eventualities among and between residents, staff and system. In the light of the theoretical model, acceptance and recognition of needs and inclusion of family members’ perspectives in understanding the residents’ needs and preferences are identified as important elements of personalized care. The meaning of personalized dementia care is shaped by the care assessment process and plan, staff competence and physical environment and safety. This study illuminates on some additional factors that shape the personalized dementia care dimensions, for example, quality of care is impacted by leadership, person-centered communication of staff with residents and personal and social life of staff. We discuss each of the dimensions and their related factors and issues as they have emerged from the study findings. | Discussion  Page No. 20 (Line No. (28-40); 21 (Line No. 1-3) |
| **Reviewer 2** |  |  |
| 1. Is the manuscript technically sound, and do the data support the conclusions?  The manuscript must describe a technically sound piece of scientific research with data that supports the conclusions. Experiments must have been conducted rigorously, with appropriate controls, replication, and sample sizes. The conclusions must be drawn appropriately based on the data presented.   Reviewer #2: Partly | **Conclusion**  The dimensions associated with the quality of personalized dementia care in rural aged care homes emerged from the data provided by care staff in five rural aged care homes in Queensland and South Australia, Australia. Personalized dementia care is multidimensional and can be described using a conceptual framework that incorporates dialectical relationships and events occur in aged care homes. To achieve quality care, the dimensions that are of importance for the aged care homes include resident and family centeredness, assessment and care planning, staff education and training, staff-resident interaction, work-life balance, leadership and organizational culture and physical environment and safety.  The current service delivery focusses too much on perceived generalized clinical care needs rather than specific person-centered care. The inclusion of the voice of residents and their family members in designing care plan is in need to establish personalized care practice for RwD to give them opportunity to live with a control over care service. Emphasis on the staff retention and recruitment and resource adequacy is important to improve quality of personalized dementia care in rural aged care homes. The aged care policy makers and organization leaders should consider work-life balance of staff as a key issue and respond with mentorship and formal support structures.  The study’s findings will contribute to the knowledge of dimensions of personalized dementia care. It is evident that rural aged care homes require additional commitment from the policy makers relating to resource stabilization and improvement. This study confirms the need for a holistic approach to respond to the barriers and integrate personalized care to improve the quality of life of RwD in rural aged care homes. | Conclusion  Page No. 25 (Line No – 18-35); 26 (Line No. 1-2) |
| 2. Has the statistical analysis been performed appropriately and rigorously?   Reviewer #2: I Don't Know | Not Applicable | Not Applicable |
| 3. Have the authors made all data underlying the findings in their manuscript fully available?  The [PLOS Data policy](http://www.plosone.org/static/policies.action#sharing) requires authors to make all data underlying the findings described in their manuscript fully available without restriction, with rare exception (please refer to the Data Availability Statement in the manuscript PDF file). The data should be provided as part of the manuscript or its supporting information, or deposited to a public repository. For example, in addition to summary statistics, the data points behind means, medians and variance measures should be available. If there are restrictions on publicly sharing data—e.g. participant privacy or use of data from a third party—those must be specified.  Reviewer #2: Yes | Not Applicable | Not Applicable |
| 4. Is the manuscript presented in an intelligible fashion and written in standard English?  PLOS ONE does not copyedit accepted manuscripts, so the language in submitted articles must be clear, correct, and unambiguous. Any typographical or grammatical errors should be corrected at revision, so please note any specific errors here.  Reviewer #2: Yes | Not Applicable | Not Applicable |
| 5A. Abstract methods: 1) no description of quasi-experimental approach. What was quasi-experimental in this study? | **Abstract Part 1**  Quality of dementia care improves with a personalized approach to aged care, and knowledge of the disease process and unique care needs of residents with dementia. A personalized model of care can have a significant impact on the overall organizational culture in aged care homes. However, the dimensions of personalized aged care relating to dementia often remain under-managed. We aim to explore the factors that shape the dimensions of personalized dementia care in rural nursing homes using qualitative data of a mixed-method ‘Harmony in the Bush’ dementia study. The study participants included clinical managers, registered nurses, enrolled nurses and care workers from five rural aged care homes in Queensland and South Australia. One hundred and four staff participated in 65 semi-structured interviews and 20 focus groups at three phases: post-intervention, one-month follow-up and three-months follow-up. A multidimensional model of nursing home care quality developed by Rantz et al. (1998) was used in data coding and analysis of the factors. | Abstract (Background and Methods)  Page No. 3 (Line No. 5-16) |
| 5B. Abstract methods: You state in the background that the aim to explore the factors that shape the dimensions of personalized dementia care in rural nursing homes using data from a large dementia study. In the conclusion you state Understanding the dimensions of quality of care is important to achieve an effective personalized approach in dementia care. This is confusing and does not reflect the results. You stated in the introduction that you want to understand the dimensions of personalized care to inform an approach that is of high quality but it is mixed up in the conclusion and makes the study purpose and conclusion disjointed. | **Abstract Part 2**  key themes including seven dimensions emerged from the findings: resident and family [resident and family centeredness, and assessment and care planning]; staff [staff education and training, staff-resident interaction and work-life balance]; and organization [leadership and organizational culture, and physical environment and safety]. A lack of consideration of family members views by management and staff, together with poorly integrated, holistic care plan, limited resources and an absence of ongoing education for staff, resulted in an ineffective implementation of personalized dementia care. Understanding the dimensions and associated factors may assist in interpreting the multidimensional aspects of personalized approach in dementia care. Staff training on person-centered approach, assessment and plan, and building relationships among and between staff and residents are essential to improve the quality of care residents receive. | Abstract (Results and Conclusion)  Page No. 3 (Line No. 18-35) |
| 5C. Introduction: 1) First line is confusing. Should it state: personalized care is integral to the quality of dementia care”? because later in the paragraph you state: “The literature indicates a personalized approach, based on active listening, recognition, compassion, attentiveness and sensitivity to the resident’s needs and preferences in providing care, promotes quality of life for residents with dementia” | Personalized care is integral to the quality of residential dementia care [1, 2]. | Introduction  Page No. 5 (Line No. 2) |
| 5D. Introduction: What previous work has been done on “dimensions of personalized aged care”? | Several studies investigate the factors and issues of personalized aged care or nursing home care such as, Rantz et al. (1998), Baldwin et al. (2013), Edvardsson, Sandman and Borell (2014) and Dyer e al. (2018); but the dimensions of personalized dementia care remain under-assessed. | Introduction  Page No. 5 (Line No. 9-11) |
| 5E. Theoretical Background: 1) Consider making a figure or table of the text describing the multidimensional theoretical model developed by Rantz et al. to better present this model. The text is too dense and hard to follow | **Table 1.** Dimensions of multidimensional nursing home care model **Dimensions** \| **Operational areas**; Central focus \| Acknowledge the needs of residents and importance of their family members perspectives; Interactions \| Communication between residents and staff; Milieu \| Overall situation and sensations of residential aged care homes such as calm and friendly place; Environment \| Aged care centers consist of various components such as room, furniture and decoration; Care \| Staff should have good understanding about each residents’ healthcare and wellbeing needs; Staff \| Knowledge and skills of staff; Safety \| Residents must feel safe, secure and free to talk with staff and obtain support when they are in need | Material and methods ˃ Theoretical background  Page No. 6 |
| 5F. Materials and Methods: 1) Again, where is the quasi-experimental approach and description? | We have removed the word ‘quasi-experimental approach’. This study reports the demographic and qualitative data of Harmony in the Bush project. Necessary changes made in the manuscript | Abstract  Page No. 3  Materials and methods  Page No. 6 |
| 5G. Materials and Methods: 2) If this is a secondary analysis of qualitative data please state this and how the data was appropriate for your research question/aim if this study. You state in the intro that data are from the Harmony in the Bush project then below that state the data are part of a larger project. It is not clear if the study is designed to answer your question or is you decided post-hoc to ask this question. This does have implications for the design and method being appropriate for the question and you will need to add justification as to why this approach is appropriate. | We like to clarify that this is not a secondary analysis of data. Harmony in the Bush project is designed to understand the personalized care support for RwD in Australian rural aged care homes and trials a personalized dementia care program. Also this study seeks to explore the factors and issues impacting on the quality of personalised dementia care in rural aged care homes. | Not applicable |
| 5H. Data Collection: 1) Vert good attention to ethics approval and staff protection! | Information sheet along with consent form were emailed to the facility managers for distribution among staff. Participants were free to contact the investigators if they wished to be interviewed and or to participate in focus group. They were also informed that they could choose to withdraw at any time of the project and no adverse effects would be on their care provision, either immediately or in the future. | Materials and methods ˃ Data collection; Page No. 7 (line No. 7-11) |
| 5I. Data Collection: 2) How/when did you know you have collected enough data to come to the themes that emerged? | In this study, data saturation was achieved in 45 interviews and 14 focus groups as the answers to the questions became repetitive at these points. However, the ongoing interviews and focus groups at different time-points continued to explore new insights. | Materials and methods ˃ Data collection  Page No. 7 (Line No. 25-27) |
| 5J. Data analysis: 1) confusing when multiple terms and processes related to the analysis (automatic search, codes, nodes, topic coding, derivative categories, provisional concept map, themes, sub-themes, categories) are used but not shown how one analytic step informed another. Consider using consistent terms and/or clearly defining how each of these analytic steps led to the next. As of now it is not clear and that might be because you are using so many undefined terms. It makes the reader a little hesitant to believe the findings because it is not clear how you got from data collection to findings. | Two researchers were involved in the data analysis process and each of them started with importing the data into a qualitative data analysis software NVivo 12 independently. We used the thematic analysis technique of Braun and Clarke (2006, 2014) in coding and analyzing the data that included several steps [19, 20]. An initial process of listening to the audios and reading the transcripts was undertaken to be familiarized with the data. The second step was open coding that provided an insight of each phenomena related to the personalized dementia care. A ‘topic coding’ was conducted as third step to search the patterns of codes across interview/focus groups’ questions. The fourth step involved a categorization of codes into nodes or candidate sub-themes. In next step, we reviewed the candidate sub-themes based on the context and generated candidate themes. Finally, sub-themes and themes were defined and presented. | Materials and methods ˃ Data analysis  Page No. 7 (Line No 30-35); 8 (Line No. 1-7) |
| 5K. Data analysis: 2) You do a nice job attending to potential bias but you might use a reference to support the approach “maintain reliability and validity of the findings” one is: Creswell J. Chapter 10: Standards of quality and verification. Qualitative Inquiry and Research Design: Choosing Among Five Traditions. Thousand Oaks, CA: Sage; 1998:193-218. | In order to maintain reliability and validity of the findings, the themes emerged from each of the researcher’s coding and categories were discussed and reviewed in the ‘Harmony in the Bush’ project’s weekly meetings [41].  Creswell J. Chapter 10: Standards of quality and verification. Qualitative Inquiry and Research Design: Choosing Among Five Traditions. Thousand Oaks, CA: Sage; 1998:193-218. | Materials and methods ˃ Data analysis  Page No. 8 (Line No. 9)  Reference List  Page No. 29 |
| 5L. Findings 1) Born in overseas- do you have an idea of where? Even if you can break this down by continent since it is close to half of the sample. | It is important to note that 37% of the total staff were from overseas including Philippines (11%), India (8%) and New Zealand (5%); while 31% of them did not speak English at home. | Findings ˃ Baseline Demographics  Page No. 8 (Line No. 18) |
| 5M. Findings 2) Wow- 37% oversees and 73% part-time and casual staff, How does this impact your findings? Maybe touch on in the discussion. | Fear of workload increase because of a low number of permanent full-time staff [73% of staff were found work part-time or casual in this study]  About 37% staff were from overseas who seem to have lack of knowledge of personalized dementia care as a formal medical condition. | Discussion  Page No. 21 (Line No 23-24)  Discussion  Page No. 23 (Line No. 2-3) |
| 5N. Findings  3) The findings seem to fall into three larger themes around issues with: family/resident, staff, and organization. | The major themes and sub-themes that emerged from the analysis of the qualitative data included: resident and family [resident and family centeredness, and assessment and care planning]; staff [staff education and training, staff-resident interaction, and work-life balance]; and organization [leadership and organizational culture, and physical environment and safety]. The explanation of the themes including the excerpts of the participants are presented in the following sections. | Findings ˃ Dimensions of personalised dementia care  Page No. 9 (Line No. 2-7) |
| 5O. Theme: Family/resident, Subthemes: assessment/care planning, resident and family centeredness    Theme: Staff, Subthemes: work life balance, staff resident interaction, staff education/training  Theme: Organization, Subthemes: physical environment, leadership and organizational culture This makes me hesitant to believe that data analysis was fully completed and there may be another step in the analysis. | Resident and family  The first main theme presented the staff perspectives of the provision of care to RwD in rural nursing homes. Dementia care was related to a lack of recognition of each resident’s care needs and exclusion of family members’ perspectives. Assessment and care planning meant a generalized approach for the residents.  Staff  The second main theme ‘staff’ described a lack of access for staff to ongoing education and training opportunities, and this was related to an isolation of RwD in staff-resident communication. Difficulty in managing professional and personal life influenced the performance of staff.  Organization  The third main theme ‘organization’ contextualized the role of leadership, staff relationships and physical environment of rural nursing homes in ensuring personalized care for RwD. | Findings ˃ Dimensions of personalised dementia care  Page no. 9 (Line No. 8-11)  Findings ˃ Dimensions of personalised dementia care  Page No. 12 (Line No. 30-33)  Findings ˃ Dimensions of personalised dementia care  Page No. 16 (Line No. 4-6) |
| 5P. Discussion Figure 1 is excellent and I think with additional analysis it can provide a useful model for designing interventions to improve person centered-ness dementia care. | Revised the Figure according to reviewer’s suggestions | Discussion  Page No. 22 |
| 6. PLOS authors have the option to publish the peer review history of their article ([what does this mean?](https://journals.plos.org/plosone/s/editorial-and-peer-review-process#loc-peer-review-history)). If published, this will include your full peer review and any attached files.   If you choose “no”, your identity will remain anonymous but your review may still be made public.  **Do you want your identity to be public for this peer review?** For information about this choice, including consent withdrawal, please see our [Privacy Policy](https://www.plos.org/privacy-policy).  Reviewer #1: No  Reviewer #2: No | Yes | Not applicable |
